# A Systematic Review of the Long-Term Outcomes of Surgical Versus Non-surgical Management for Types A3 and A4 Thoracolumbar Spinal Fractures With No Neurological Deficits

**DOI:** 10.7759/cureus.72620

**Published:** 2024-10-29

**Authors:** Sultan A Alfaedi, Abdullah M Alharbi, Abdulrahman S Hassan, Faris A AlZahrani, Jawad Albashri, Ahmed S Albashri, Anas Alqahtani, Mohammed Hariri

**Affiliations:** 1 Spine Surgery, King Abdullah Medical Complex – Jeddah (KAMCJ), Jeddah, SAU; 2 Orthopedic Surgery, King Abdulaziz University Hospital, Jeddah, SAU; 3 Medicine and Surgery, University of Jeddah, Jeddah, SAU; 4 College of Medicine, University of Jeddah, Jeddah, SAU

**Keywords:** a3 fracture, a4 fracture, long-term clinical outcomes, non-surgical management, surgical management, thoracolumbar fractures

## Abstract

Thoracolumbar spinal fractures remain a significant concern in the orthopedic and neurosurgical practice and have a risk of neurological deficits in patients. However, in the majority of the cases, the neurological status remains intact. Generally, conservative management is favored for fractures of low severity and the surgical option is reserved for severe fractures. The management of types A3 and A4 thoracolumbar spinal fractures without neurological deficits still remains debatable. This systematic review aims to compare long-term outcomes of surgical versus non-surgical management in the treatment of types A3 and A4 fractures with no neurosurgical deficit. A systematic search was undertaken in various databases including PubMed, Scopus, and Web of Science to identify relevant studies. The inclusion criteria focused on patients with types A3 and A4 thoracolumbar fractures without neurological deficits. Studies published after 2010 and having a minimum follow-up period of two years were considered for inclusion. The risk of bias was assessed using the Newcastle-Ottawa Scale. A total of 1,973 potential studies were identified, out of which three studies were included in the systematic review. The mean follow-up duration of the studies was more than 2.5 years. A total of 141 patients were included in the studies, with 65 patients in the conservative and 76 patients in the surgical group. Out of three studies, two studies favored surgical management for better functional outcomes, while one study suggested better long-term outcomes with conservative management. Regarding complications, no significant complications were reported in both groups and there was no significant difference between the groups. The risk of bias was low across most studies. Although two of the three studies included in the systematic review favored the surgical approach in the management of types A3 and A4 fractures, there is still a lack of conclusive evidence that favors either approach.

## Introduction and background

Thoracolumbar spinal fractures represent a significant challenge in orthopedic and neurosurgical practice. These fractures mostly result due to high-energy trauma such as motor vehicle accidents or falls from height. Thoracolumbar fractures (T11-L3) account for approximately 90% of all the spinal fractures [1]. About two-thirds of the thoracolumbar fractures include vertebral body compression without dislocation or ligament damage. These fractures are classified as AOSpine type A. AOSpine classification categorizes thoracolumbar fractures into distinct types based on the mechanism of injury and the resultant anatomical damage [2,3]. AOSpine type A0, A1, and A2 are less severe fractures whereas A3 and A4 are more severe fractures and involve the posterior wall of the vertebral body. A3 fractures involve the posterior wall of one vertebral plateau, while A4 fractures affect both plateaus [4]. Approximately 20% of all fractures include compression fractures involving comminution of the vertebral body [5,6]. Loss of neurological function is a significant concern in patients with thoracolumbar fractures. Thoracolumbar burst fractures lead to loss of neurological function in around half of the patients [4].

The involvement of the posterior wall, as seen in A3 and A4 fractures, causes the bone fragment to move backward into the vertebral canal, which can result in neurological deficits in approximately 15% of cases [7,8]. Despite the absence of neurological deficits in some patients, these fractures can lead to long-term complications if not appropriately managed, including chronic pain, kyphotic deformity, and impaired function. Currently, there is consensus regarding the management of less severe AO spine-type fractures which involves conservative treatment options. Similarly, types B and C fractures regarded as severe fractures and cases with neurological deficits are managed with surgical interventions [5]. Despite the fact that most of the patients with A3 and A4 fractures maintain neurological functions, their treatment still remains controversial. The primary aim of the treatment in A3 and A4 fractures is to ensure early mobility, avoid kyphotic deformity, and safeguard neurological function [9]. Surgical treatment usually has superiority over conservative management as it provides immediate stabilization, early mobility, independence from orthotics, and early mobility [6]. However, there is the risk of complications as well, such as infection, implant failure, and arthrosis [10]. On the other hand, conservative treatment is associated with high morbidity due to the extended period of absolute rest, which usually lasts from six to eight weeks. This approach has shown promising functional outcomes, minimal progression of deformities, and a low rate of neurological decline [10,11].

Previously, some systematic reviews and meta-analyses have investigated the difference between conservative and surgical treatments in the management of A3 and A4 fractures [4,12]. However, there are certain limitations. First, they included studies that had both short-term and long-term outcomes. Second, most of the included studies were old. Therefore, the present systematic review aims to fill this gap in the literature. We aimed to investigate long-term outcomes, with a minimum follow-up of two years, and included the latest evidence.

## Review

Methodology

The present systematic review was conducted according to the guidelines of the Preferred Reporting Items for Systematic Reviews and Meta-Analyses (PRISMA) guidelines [13]. The PICOS framework for this systemic review was as follows: patients (P), patients with types A3 and A4 thoracolumbar spinal fractures and no neurological deficits; intervention (I), surgical management (e.g., instrumented spinal fusion); comparison (C): non-surgical management (e.g., bracing, early mobilization); outcomes (O): long-term clinical outcomes (e.g., pain, disability, return to work), radiological outcomes (e.g., kyphosis, loss of vertebral body height), and incidence of complications; study design (S): randomized controlled trials, prospective, and retrospective cohort studies.

Search Strategy and Data Sources

A systematic search was carried out in various databases to find relevant studies regarding the long-term outcomes of surgical versus non-surgical management for types A3 and A4 thoracolumbar spinal fractures with no neurological deficits. Databases such as PubMed, Scopus, and Web of Science were searched for the studies. The keywords used for the search included “surgical procedures”, “A3 fracture”, “A4 fracture”, “neurological deficit”, and “neurological function”. Boolean operators “OR” and “AND” were used for combining the keywords. Both MeSH terms and title/abstract were used for the search. Google Scholar and the reference section of the potential studies were also explored to identify more studies. The inclusion criteria of this systematic review included 1) studies involving patients with types A3 and A4 thoracolumbar spinal fractures and no neurological deficits; 2) studies comparing the long-term (minimum two-year follow-up) clinical and radiological outcomes of surgical and non-surgical management; 3) randomized controlled trials, prospective, and retrospective cohort studies; and 4) studies published in English. The exclusion criteria comprised of 1) studies with a follow-up duration of less than two years; 2) studies involving patients with neurological deficits; 3) case reports, case series, and review articles; and 4) studies published in languages other than English.

Data Extraction 

The database search results were retrieved and merged into a reference manager (EndNote® X7). After merging the results, the file was transferred to Rayyan (Rayyan Systems Inc, MA, EUA), a software designed for the screening of studies [14]. First, the duplicates were removed from the included studies. During the screening process, two independent reviewers (AB and MU) were involved. To reduce the risk of bias, the "blind" was turned on in the Rayyan. In the first step, studies were selected based on the title and abstract of the studies. Second, the blind was removed, and conflicts were resolved after discussion. In case of any disagreements, a third reviewer (AY) was involved, and the decision was made based on the majority opinion. Third, a full-length screening was performed, and the final decision was made about the inclusion. All relevant information from the included studies such as demographic details, outcomes, and complications were retrieved in an Excel file.

Risk-of-Bias Assessment

The risk of bias was performed by two authors. The Cochrane Risk of Bias tool was used to assess the methodological quality of included randomized controlled trials, and the Newcastle-Ottawa Scale was used for observational studies.

Results

Included Studies

**Figure 1:**
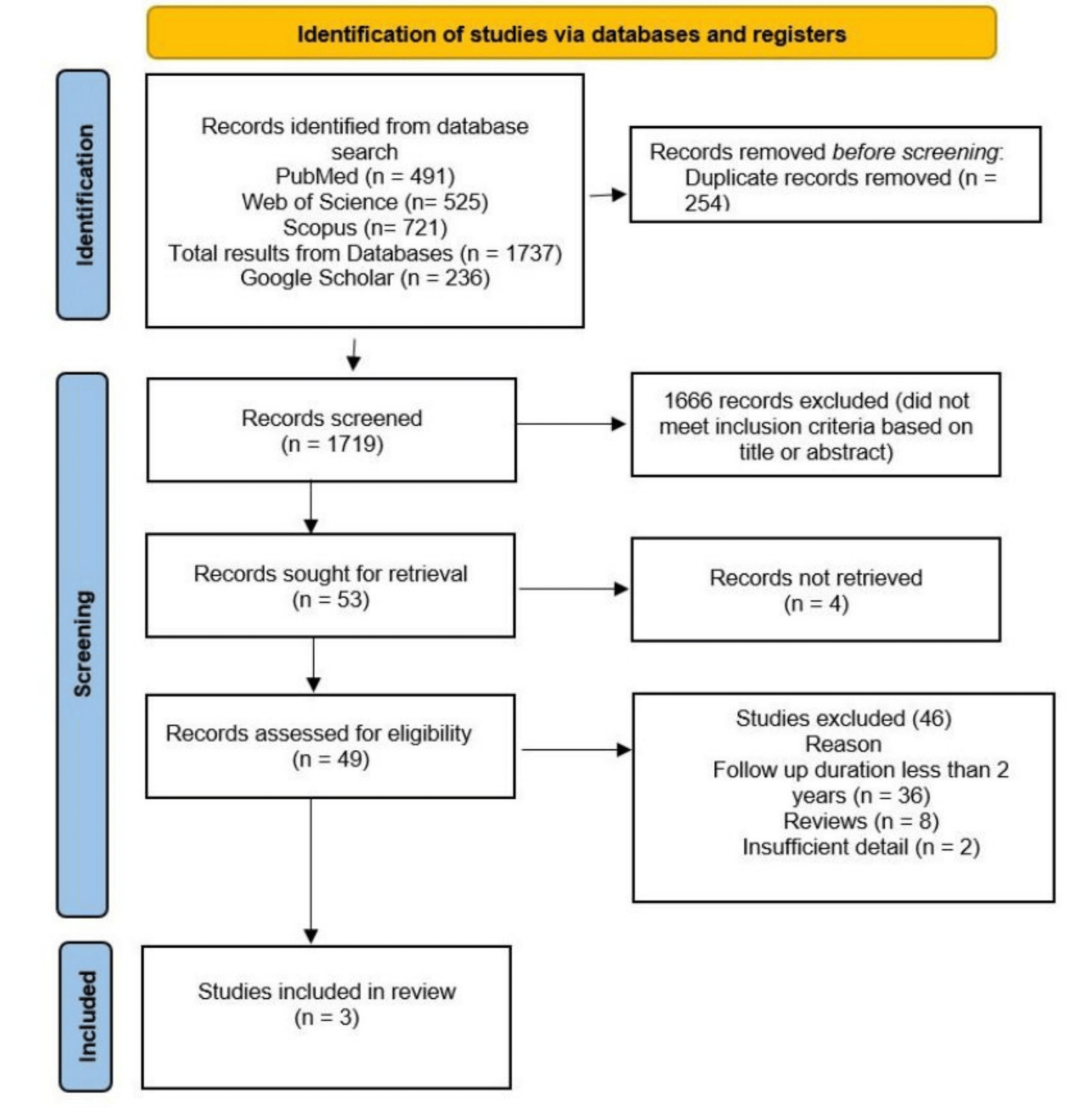
Preferred Reporting Items for Systematic Reviews and Meta-Analyses (PRISMA) flow diagram of the systematic review.

The systemic search yielded 1.973 studies (PubMed = 491, Web of Science = 525, and Scopus = 721). A total of 236 studies were retrieved from Google Scholar. After removing duplicates, 1,666 records were removed during the screening process based on the title and abstract. A total of 53 studies were assessed during full-length screening. Only three studies met the inclusion criteria and were included in the systematic review. The PRISMA flow diagram is shown in Figure 1.

Risk of Bias

The risk of bias, measured with NOS, revealed a quality score between 7 and 9 (Table 1). In all of the included studies, the risk of bias was low. Two studies had a risk of bias regarding comparability [6,15].

**Table 1:** Risk of bias measured with the Newcastle Ottawa Scale (NOS)

	Selection				Comparability		Outcome			
Study	Representativeness of the exposed cohort	Selection of the non-exposed cohort	Ascertainment of exposure	Demonstration that outcome of interest was not present at the start of study	Controls for the most important risk factors	Controls for other risk factors	Assessment of outcome	Was follow-up long enough for outcomes to occur	Adequacy of follow-up of cohorts	Total quality score
Vialle et al., 2023 [16]	1	1	1	1	1	1	1	1	1	9
Pehlivanoglu et al., 2020 [15]	1	1	1	1	0	0	1	1	1	7
Wood et al., 2015 [6]	1	1	1	1	0	0	1	1	1	7

Study Characteristics

A total of three studies met the inclusion criteria and were included in this systematic review. Out of the included studies, one was a prospective observational study; one was a retrospective, comparative, non-randomized study; and one was a prospective randomized study. A total of 141 patients were included in the studies, with 65 patients in the conservative and 76 patients in the surgical group. The minimum follow-up period in all of the studies was above 2.5 years. All of the included studies compared the outcomes of surgical and non-surgical approaches for treating A3 and A4 thoracolumbar spinal fractures without neurological deficits. When results were compared for surgical and non-surgical management, two studies showed that surgical management provides better functional outcomes than non-surgical management [15,16]. One study supported non-surgical management and suggested that it provides better outcomes on long-term follow-up [6]. No complications related to surgical and non-surgical management were reported in these studies.

**Table 2:** Summary of the included studies DWS: Denis Work Scale, EQ-5D: EuroQol-5D, RMDQ: Roland Morris Disability Questionnaire, NRS: numeric rating scale, QOL: quality of life, VAS: Visual Analog Scale, ODI: Oswestry Disability Index, JOA: Japanese Orthopaedic Association

Author and year	Study design	No. of participants in each group	Age	Follow-up period	Outcome assessment methods	Outcomes	Complications	Conclusion
Vialle et al., 2023 [16]	Prospective observational study	Conservative= 23 Surgical group= 36	Patients with age <18 and >65 years	Up to 2.5 years	NRS, RMDQ, EQ-5D, DWS	No statistical difference was observed in functionality (RMDQ: p> 0.05), pain (NRS: >0.05) QOL, and return to work	No grade-worthy or severe complications were observed	Both options are viable and possess equivalent results. There was a tendency towards better clinical results in surgical treatment of A4 fractures.
Pehlivanoglu et al., 2020 [15]	Retrospective, comparative, non-randomized, single-center trial	Surgical = 21, Conservative = 24	Mean age surgical = 34.3, Mean age conservative = 45.7	Mean follow-up surgical = 63.1 months, Mean follow-up conservative = 67.1 months	ODI, VAS, JOA	Surgical management provides better radiological outcomes than conservative management. No statistically significant difference was observed in terms of clinical outcomes and quality of life.	No complications	Better radiological outcomes have been observed with surgical management while clinical outcomes are the same for both groups
Wood et al., 2015 [6]	Prospective randomized study	Surgical = 19, Conservative = 18	Median age surgical = 62, median age non-surgical = 62.5	Median follow-up surgical = 216 months, median follow-up non-surgical = 229.5 months	VAS, ODI, RM	The difference in pain scores at long-term follow-up was statistically significant. A statistically significant difference was observed in the Oswestry questionnaire and RMDQ scores.	N/A	Non-surgical management provides better functional outcomes than surgical management

Discussion

The findings of the present systematic review showed that surgical treatment may present better outcomes compared to conservative management in type A3 and A4 fractures. However, due to the paucity of studies that assessed long-term outcomes, the findings are not conclusive. The findings of the present systematic review are consistent with some of the previous systematic reviews and meta-analyses, which were also not able to draw conclusions between conservative and surgical approaches. One of the major reasons for this was the small number of studies included in these meta-analyses. Gnanenthiran et al. included only four studies while Abudou et al. included only two studies [17,18]. Abudou et al. demonstrated that the evidence is insufficient to say which treatment was better for spine fractures. However, surgery is likely to be associated with some more complications and may need subsequent surgeries and greater initial costs [18]. Gnanenthrian et al. found no difference in baseline pain, RMDQ scores, and kyphosis between the conservative and surgical groups. Operative management was associated with improved residual kyphosis but could not improve function or pain at an average of four years after injury. Moreover, operative management was associated with high complication rates and costs [17]. Similarly, the present review did not reveal the best treatment option due to insufficient evidence regarding the superiority of both surgical and non-surgical treatment options for patients with thoracolumbar fractures without neurologic deficits.

Two recent systematic reviews have also been published on the topic. Chou et al., in their systematic review and meta-analysis, suggested that surgical treatment is unlikely to be considered a priority because of its low superiority over conservative treatment in burst fractures in neurologically intact persons. In the six-month follow-up, minimally invasive processes such as vertebroplasty or percutaneous transpedicular fixation can be beneficial in terms of ODI and VAS scores. Despite the lack of apparent superiority of the surgical approach in the long term, the role of surgery in the management of A3 and A4 fractures still remains crucial [12]. Another systematic review by Rometsch et al. investigated the efficacy of surgical and non-surgical treatment approaches for A3 and A4 thoracolumbar fractures in patients without neurological deficits. A total of 12 studies were included in this review, four of which compared the conservative and surgical treatment methods. This study found no difference in the pain and disability outcomes between the groups in A3 and A4 thoracolumbar fractures in neurologically intact patients. This study was not able to provide a comparative analysis of the radiological results in these groups due to the lack of uniform reporting, though previous studies have shown a possible benefit of surgical treatment of A3 and A4 fractures with regard to radiographic outcomes [4].

Due to the lack of consensus on the management of A3 and A4 thoracolumbar spinal fractures without neurological deficits, this topic still remains debatable. Some authors suggest that conservative management should be employed in those cases with less degree of vertebral compression and minor kyphosis [19]. One of the studies in the present systematic review points toward better functional outcomes in the non-surgical or conservative treatment method compared to the surgical treatment on long-term follow-up [6]. These results have been supported by a previous study performed by Shen et al., which suggested that the surgical group had less pain compared to the non-surgical group at three-month follow-up and improved Greenough Low Back Outcome Score at the six-month evaluation; however, the outcomes were similar at the two-year follow-up [20]. The efficacy of conservative treatment methods has also been established in a retrospective review conducted by Tropiano et al. [21]. Their findings suggested that closed reduction and casting are safe and provide acceptable functional outcomes in the patients. At the eight-month follow-up, 64% of patients had no pain and 58% had employment [21]. Furthermore, conservative treatment results in a lower number of complications as compared to surgical treatment. These complications include sepsis, pneumonia, wound infections, and urinary tract infections [22].

Due to the instability of thoracolumbar spinal fractures, surgical treatment methods are often recommended. Surgery is mostly suggested as these fractures involve various neurological complications [23]. In the present systematic review, two studies support using surgical methods for treating thoracolumbar spinal injuries without neurological deficits. In a prospective observational study by Vialle et al., it was concluded that although both surgical and non-surgical options are viable, surgical methods offer better clinical results. In their study, Pehlivangolu et al. revealed that surgical methods provide better radiological outcomes on long-term follow-up than non-surgical methods. Previously, Siebenga et al. also emphasized the importance of using surgical methods for A3-type thoracolumbar spinal fractures [10]. At a 4.3-year follow-up duration, kyphotic deformity incidence was significantly lower in the surgical group compared to conservative management. Furthermore, pain outcome measures and patients being re-employed were also significantly improved in the surgical group compared to the conservative group [10]. Muratore et al., in their retrospective study, also supported that the surgical approach is valid for thoracolumbar fractures [24].

Strengths and limitations

This is the first systematic review that investigated the long-term outcomes (minimum of two years) of surgical versus conservative approaches for the management of A3 and A4 fractures. For this systematic review, several databases were explored to identify studies. There are several limitations as well that can impact the findings of this systematic review. First, due to the strict inclusion criteria (studies published after 2010 and minimum follow-up of two years), only limited studies were identified in the literature which did not reveal conclusive evidence in favor of any treatment approach. Furthermore, none of the included studies were randomized controlled trials and all were observational studies.

Implications for practice and research

The evidence obtained in the present systematic review slightly favors the surgical approach regarding improvement in functional outcomes; however, due to the paucity of the evidence the findings are inconclusive, only three studies with a minimum follow-up of more than two years have been published so far. Therefore, there is a need for more high-quality evidence from RCTs to investigate the radiological and functional outcomes in surgical and conservative treatment approaches in type A3 and A4 fractures. Furthermore, the follow-up duration is usually not that long to identify long-term outcomes. Future studies should focus on at least more than two years of follow-up duration.

## Conclusions

The findings of the present systematic review yielded mixed results, with some studies favoring the surgical approach and one study favoring conservational management. There is a paucity of studies that have long-term follow-up periods, with a minimum duration of more than two years. Due to this reason, the present systematic review was unable to achieve conclusive evidence in favor of any approach. Regarding complications, both studies show no significant complications or there was no difference in the complications in compared groups. The heterogeneity of the results can be attributed to variations in design, follow-up duration, and outcome measures. The findings of the systematic review have significant clinical and research implications as there is a need for large RCTs, with longer follow-up duration to assess the functional and radiological outcomes in surgical and conservational management in type A3 and A4 fractures. 
